# Vienna Letter

**Published:** 1880-06

**Authors:** W. T. Belfield

**Affiliations:** Vienna


### Foreign Correspondence.

---

ARTICLE VIII.

VIENNA, April 24, 1880.

MESSRS EDITORS:—The medical visitor in Vienna loses no time in seeking the great hospital which makes this city the Mecca of the medical world, attracting hither, as it does, knowledge-seekers not simply from America, North and South, but from all Europe, even England and France. Permit me to anticipate right here, and say that while I have as yet no personal acquaintance with the professional advantages in the other European capitals, I am assured by men who have studied in London, Paris, Berlin and Leipsig, that the facilities here surpass all others. One gentleman, after a thorough trial of Paris and Berlin, came to Vienna and was so pleased that he has remained here three years; another, after studying here a while, went to Paris, but returned to Vienna in less than three months. An English gentleman here acknowledged that while the London hospitals were more elegant, and many of the teachers, in his opinion, as renowned, yet the material there is not nearly so accessible as here—the London hospitals being scattered all over and around that immense metropolis, while in Vienna, all the instruction, clinical and didactic, is given within five minutes' walk of the hospital gate.

One approaches the hospital without any suspicion of its beneficent character; it is a bleak, dingy, stuccoed affair, bordering the sidewalk of a narrow street; it might be a hotel, a wholesale business house, a prince's palace or a private dwelling, for in Vienna these are all alike unattractive and forbidding in their external appearances. But the legend over the portal—" Salus et Solatio Ægrorum—Franciscus I., MDCCCXXXIV"—reveals to me the fact that this is the way we long have sought. Passing the gate, we enter a square court or park, about two acres in extent, enclosed by the hospital pavilions and rich in shrubbery, grass, trees, fountains, etc. The graveled walks of this very attractive place are lined with settees, occupied largely by the patients, male and female, who are loosely clad in a grotesque attire of striped linen, strongly suggestive of a circus clown or a penitentiary convict. A little exploration discloses several such courts, all enclosed by hospital pavilions, comprising in all perhaps twenty acres. The buildings themselves are old, dingy and devoid of many of the modern improvements—speaking tubes, electric signals, elevators, etc. They are generally, however, well heated, fairly ventilated and *scrupulously clean.* In the surgical pavilions we find at any time one or more wards empty and in process of renovation—scraping and whitewashing of walls, etc. The pavilions are under the jurisdiction of the respective professors, each of whom has his clinic-room connected with his wards.

Here we find not only 2,500 patients, but also an array of renowned instructors; and the question arises, shall we first visit Billroth or Bamberger, Brüeke or Stricker, Hebra or Neumann, Carl Braun or Spath, Stellwag or Politzer? As conductors of the expedition, I shall guide the JOURNAL reader first to Billroth, who heads the list alphabetically and perhaps otherwise, also.

We enter a room provided with seats for 250 persons, the center of which is an arena perhaps fifteen feet square. In the middle of this stands a revolving operating table, while around the sides are arrayed several cases full of glittering instruments. Suspended on the wall are two reservoirs marked respectively three per cent. and one per cent. (carbolic acid solution), extending from which into the arena are two rubber tubes terminating in stop-cocks. Several assistants in clean linen dusters, are busy preparing, while thirty or forty students lounge on the narrow, straight-backed, antiquated benches. About half an hour after the hour appointed for the opening of the clinic (9 a. m.), the hum of conversation is suddenly hushed, and there enters the Herr Hofrath and Professor Theodor Billroth—a man of fine presence, distinguished by a well-formed head, benevolent countenance and prodigious abdomen. Without any formality he begins to speak, but unfortunately in a tone so low that his words are audible at the distance of only a few feet; the difficulty in hearing him is increased by his habit of sauntering around the arena and of occasionally seating himself in a chair with his back to as many auditors as possible. As a result, the attention of those present is bestowed upon books, papers, conversation, etc., and the great surgeon talks to perhaps a dozen listeners.

Meanwhile, his ten assistants, in the regulation linen duster, are preparing a patient—a process worthy of our attention. The anæsthetic is a mixture of chloroform, ether and alcohol; the inhaler a mere strip of canton flannel stretched over a metallic frame, held an inch or so above the nose and mouth. Only a few drops are administered at once, and the pulse is carefully observed by an assistant, who does nothing else during the entire operation. Meanwhile, another assistant having carefully washed his hands, exposes the part to be operated upon, shaves it carefully—even if it be a woman's cheek—scrubs it thoroughly with a nail brush, soap and water, deluges it with carbolized water, and then covers the adjacent parts with towels saturated with carbolized solution. A fourth assistant, during this interval, selects the instruments to be used and immerses them also in a carbolized solution; a fifth plunges about thirty sponges, previously washed and carbolized, into a jar of the same solution—a large number being required even for a trivial operation, because a sponge is used but once, and then re-carbolized. Finally, Billroth takes a scalpel and proceeds with great care and deliberation to make the necessary operation, occasionally stopping to explain to the class various points of interest. One of the most interesting features of the performance is the watchfulness and alacrity of his numerous assistants; they anticipate all the operator's wants; the needed instrument is at his elbow when he wants it; a sponge is constantly hovering over the wound, and yet everything is done quietly and smoothly, Billroth himself setting an example of courtesy and good humor toward all.

Every bleeding vessel, small as well as large, is temporarily closed by means of a "bull-dog" forceps, and finally tied with catgut. At the conclusion of the operation, the lips of the wound are approximated by silver wires fastened with lead-plates, and then fixed in exact apposition by silk sutures set very close together, space being left only for several large drainage-tubes, which are tightly hugged by the lips of the wound, cut off close and fastened with safety-pins. The usual antiseptic dressing is then applied ; great pressure is always made, the elastic bandage being frequently called into requisition for that purpose, applied, of course, outside of the gauze.

No reference has been made to the carbolized spray. As a skeptic in regard to the antiseptic theory, I am pleased to state that Billroth has discarded his atomizers, or rather has determined not to employ them until convinced by results that they are desirable. The other surgeons here have done likewise—I have not seen the antiseptic spray used in Vienna. Yet, as the reader has observed, he retains the undeniably valuable feature of Listerism—cleanliness—and has thus far secured all the good claimed for the Listerian system. The valuable points in Billroth's practice appear to be : 1, the careful ligation of all blood-vessels ; 2, the accurate adjustment of the lips of the wound ; 3, the compression of the wound. I have seen a dressing removed five days after a bloody operation on the face, and the wound showed no sign of suppuration, the edges entirely free from inflammatory exudate. Union by first intention is an ordinary result, even for some amputations.

Their management of fractures and their mechanical surgery in general is, in my observation, inferior to ours. They seem to lack much of the mechanical ingenuity and tact which characterize our surgeons—dressing fractures of the thigh solely by extension, *a la* Buck, for example. Yet it must be confessed that their results compare not unfavorably with ours, even in this respect. They have one advantage in the apparent fortitude of their patients, who endure suffering and inconvenience with wonderful equanimity. I say apparent fortitude, for the real explanation may lie in their insensitive nervous systems rather than in their self-control. I have seen an operation on the face continue three-quarters of an hour, during the last thirty minutes of which no anæsthetic was administered, and yet no complaint was heard from the patient.

The amount of surgical material here is immense, as may be inferred from the fact that Billroth and Dumreicher give each a daily clinic from two to two and a half hours in duration, or from the other fact that Billroth recently performed eight ovariotomies in one week.

These clinics are but one of the valuable features of surgical instruction here. The chief assistants, three in number, give private courses in minor surgery, diagnosis and treatment of fractures and dislocations, applications of dressings, etc., to illustrate which they have access to all the surgical wards. They give also valuable courses in operative surgery on the cadaver—first performing the various operations—ligation of arteries, amputations, excision of joints, herniotomy, lithotomy, etc., before the class, and then superintending the performance of the same by the various students. Such a course costs about eight dollars, and requires sixty to seventy hours, during which each student performs every operation under the personal direction of the instructor. Such instruction is, of course, possible only where material is cheap and abundant, and this is eminently true of Vienna, where a dissecting ticket, costing $2.50, entitles a man to all the material he wishes for a *year*.

In the other departments of the hospital, in the great "Poliklinik" or free dispensary (also under the care of the university), and in the various laboratories of the college one finds equally good advantages, some of which will receive our attention later.

W. T. BELFIELD.

---

RETENTION OF LIFE IN THE NEW-BORN.—Dr. Hoffman calls attention in the *Medical Record* to a case in which the cord formed a clove-hitch around the child's neck, so tight that for at least thirty minutes there was complete strangulation, yet persevering efforts at resuscitation were successful. He also speaks of other reported cases in which from one to four hours had elapsed without respiration, and life was retained.

